# Synthesis and Applications of Novel Low-Dimensional Nanomaterials in Catalysis

**DOI:** 10.3390/molecules31030482

**Published:** 2026-01-30

**Authors:** Lu Lu

**Affiliations:** Paris Curie Engineer School, Beijing University of Chemical Technology, Beijing 100029, China; lulu@mail.buct.edu.cn

Low-dimensional nanomaterials have attracted substantial interest as promising candidates for heterogeneous catalysis thanks to their high density of exposed active sites and abundant unsaturated coordination atoms [1,2,3,4]. Over the past decade, there has been considerable progress made in improving the catalytic performance of these nanomaterials in heterogeneous reactions. At the nanoscale, catalytic behavior is strongly influenced by the atomic structure and electronic properties of a material [5]. For instance, in two-dimensional (2D) layered nanomaterials, charge carrier transport is primarily confined to the in-plane direction, while individual layers are bound by van der Waals interactions, leading to atomic-scale thickness such as graphene and transition metal dichalcogenides [6,7,8]. One-dimensional (1D) nanostructures, including nanorods, nanotubes, and nanowires, meanwhile exhibit distinct morphological and electronic characteristics due to their confined geometry [9,10,11]. Moreover, zero-dimensional (0D) nanomaterials such as nanoparticles and quantum dots possess high surface-to-volume ratios and ultra-small dimensions, which have been demonstrated to enhance their catalytic performance in a range of reactions [12,13].

In this Special Issue, titled “Synthesis and Application of Novel Low-Dimensional Nanomaterials in Catalysis”, which I co-edited with Prof. Dr. Xingcai Wu from Nanjing University, we aim to comprehensively disseminate scientific findings related to advances in low-dimensional nanomaterials and their applications. This collection includes thirteen papers, consisting of six review articles [1,2,3,4,5,6] and seven research papers, which focus on catalytic topics related to low-dimensional nanomaterials [7,8,9,10,11,12,13].

Among the review articles, Huang et al. [Contribution 1] summarized the mechanisms of cascade oxygen reduction reactions and the synthesis of transition metal-based single-atom catalysts for cathode electrocatalysis in efficient wastewater treatment. They also discussed key factors that affect the treatment’s performance.

Zhou et al. [Contribution 2] addressed the development of metal–organic framework (MOF)-based photocatalysts for hydrogen evolution. They highlighted specific advances, such as the use of Ir (III) complexes and sandwich structures, which illustrate the improvement in their performance, while also discussing ongoing challenges such as stability and scalable synthesis, to ultimately provide a roadmap for future sustainable research.

Ling et al. [Contribution 3] provided a comprehensive overview of MOF classifications and their current applications as catalysts, catalyst supports, and membranes in fuel cells, alongside a discussion of the potential prospects and challenges related to the use of MOFs and their derivatives in this field.

Fu et al. [Contribution 4] organized and discussed the role that single-atom (SA) catalysts supported on two-dimensional (2D) nanomaterials can play in enhancing the hydrogen evolution reaction (HER). They summarized the commonly used synthesis methods, advanced characterization techniques, various types of 2D supports, and the correlation between the structure and the HER performance.

Lu et al. [Contribution 5] summarized the general synthetic strategies and structural characterization methods for dual-atom catalysts (DACs) and discussed recent experimental advances in DAC-catalyzed water-splitting reactions to provide inspiration for future research.

Bai et al. [Contribution 6] elaborated on the lattice oxygen mechanism and adsorbate evolution mechanism of the oxygen evolution reaction (OER). They summarized and discussed the recently reported RuO_2_-based OER electrocatalysts under acidic conditions and proposed some modification strategies to further improve their activity and stability.

Concerning the low-dimensional nanomaterials research papers, Cui et al. [Contribution 7] fabricated Co_3_O_4_ with abundant oxygen vacancies and exposed highly active crystal facets, demonstrating the superior OER activity compared to Co_3_O_4_ nanocubes with only (100) facets exposed. This unique hollow structure further facilitated mass transport, prevented nanosheet stacking, and exposed more edge sites for OER. Zhou et al. [Contribution 8] synthesized WO_3_@TCN, which proved effective in degrading organic dyes and enabling photocatalytic H_2_ evolution. The synergistic effect between WO_3_ and TCN, along with the porous structure of TCN and the formed heterojunction, accelerated both degradation and H_2_ generation. Zhao et al. [Contribution 9] synthesized platinum (Pt) nanoparticles with a size of less than 50 nm, and the resulting Pt/C nanoparticles exhibited remarkable electrochemical performance in the formic acid oxidation reaction (FAOR), with higher activity and stability than commercial Pt/C. Zhang et al. [Contribution 10] prepared In_2_O_3_/Bi_2_WO_6_ II-type semiconductor heterojunction composites for the photocatalytic degradation of rhodamine B. Active species experiments suggested that holes (h^+^) were the primary species. Yang et al. [Contribution 11] reported one-dimensional NiS_2_/NiS/Mn_2_O_3_ nanofibers prepared via electrospinning, and these demonstrated outstanding OER performance in an alkaline solution. Li et al. [Contribution 12] developed Au_10_Pt_1_@MnO_2_ catalysts for the methanol oxidation reaction (MOR) and comparative analysis revealed that introducing MnO_2_ nanosheets both increased reactive sites and promoted reaction kinetics. The high surface area of the MnO_2_ nanosheets facilitated charge transfer and induced modifications in the electronic structure of the composite. Zhang et al. [Contribution 13] addressed a critical methodological issue regarding the accurate evaluation of electrocatalysts, emphasizing the influence of the electrode position. They strongly recommended keeping the electrode position unchanged during the electrochemical measurement as this should ensure fair comparisons and reliable results.

We sincerely hope that the articles published in this Special Issue will contribute to further innovative and in-depth research in the field of low-dimensional nanomaterials. These materials have tremendous potential both in electrocatalysis and in photocatalysis. We expect the research presented here to play a pivotal role in addressing the key challenges in this field and to accelerate the development of next-generation catalysts with enhanced efficiency, selectivity, and stability for sustainable energy and environmental applications.
